# β-Arrestin1 and 2 differentially regulate PACAP-induced PAC1 receptor signaling and trafficking

**DOI:** 10.1371/journal.pone.0196946

**Published:** 2018-05-07

**Authors:** Yusuke Shintani, Atsuko Hayata-Takano, Keita Moriguchi, Takanobu Nakazawa, Yukio Ago, Atsushi Kasai, Kaoru Seiriki, Norihito Shintani, Hitoshi Hashimoto

**Affiliations:** 1 Laboratory of Molecular Neuropharmacology, Graduate School of Pharmaceutical Sciences, Osaka University, Suita, Osaka, Japan; 2 Center for Child Mental Development, United Graduate School of Child Development, Osaka University, Kanazawa University, Hamamatsu University School of Medicine, Chiba University and University of Fukui, Suita, Osaka, Japan; 3 Department of Pharmacology, Graduate School of Dentistry, Osaka University, Osaka, Japan; 4 Interdisciplinary Program for Biomedical Sciences, Institute for Academic Initiatives, Osaka University, Suita, Osaka, Japan; 5 Division of Bioscience, Institute for Datability Science, Osaka University, Suita, Osaka, Japan; Indian Institute of Technology Kanpur, INDIA

## Abstract

A pituitary adenylate cyclase-activating polypeptide (PACAP)-specific receptor, PAC1R, is coupled with multiple signal transduction pathways including stimulation of adenylate cyclase, phospholipase C and extracellular-signal regulated kinase (ERK)1/2. PAC1R has been shown to exert its long-lasting and potent signals via β-arrestin1 and β-arrestin2. However, the precise roles of the two β-arrestin isoforms in PACAP-PAC1R signaling remain unclear. Here we examined the interaction between the two β-arrestin isoforms and PAC1R, β-arrestin-dependent PAC1R subcellular localization and ERK1/2 activation. Upon PACAP stimulation, although PAC1R similarly interacted with β-arrestin1 and β-arrestin2 in HEK293T cells, the complex of PAC1R and β-arrestin2 was translocated from the cell surface into cytosol, but that of β-arrestin1 remained in the cell surface regions in HeLa cells and mouse primary cultured neurons. Silencing of β-arrestin2 blocked PACAP-induced PAC1R internalization and ERK1/2 phosphorylation, but silencing of β-arrestin1 increased ERK1/2 phosphorylation. These results show that β-arrestin1 and β-arrestin2 exert differential actions on PAC1R internalization and PAC1R-dependent ERK1/2 activation, and suggest that the two β-arrestin isoforms may be involved in fine and precise tuning of the PAC1R signaling pathways.

## Introduction

Pituitary adenylate cyclase-activating polypeptide (PACAP) [1] is a multifunctional neuropeptide expressed in the central and peripheral nervous systems and acts as a neurotransmitter and neurotrophic factor via three subtypes of G protein-coupled receptors: a PACAP-preferring (PAC1) receptor (PAC1R) and two vasoactive intestinal polypeptide (VIP)-shared (VPAC1 and VPAC2) receptors [1, 2]. PACAP-deficient mice [3] have implicated the signal in a variety of biological roles that include psychiatric and neurological conditions [4–6], neuropathic pain [7], actions as endogenous protective factor [8, 9] and stimulating tear secretion [10]. Accumulating evidence suggests that altered PACAP and VIP signals can be a risk factor for psychiatric and neurological disorders such as schizophrenia and stress-related disorders [11–15].

PAC1R belongs to a class B G protein-coupled receptor (GPCR) family, which couples to Gs and Gq proteins and can stimulate several transduction pathways such as adenylate cyclase, phospholipase C, intracellular calcium increase, p38 and/or extracellular-signal regulated kinase (ERK)1/2 signaling pathway [1, 2, 16, 17]. Previously, we showed that PACAP and NGF synergistically induce prolonged activation of ERK1/2 in PC12 cells [18].

The PACAP-induced ERK1/2 signaling pathway is considered to have two major pathways, the G protein-dependent signaling pathway leading to inositol phosphate (IP) accumulation and calcium release, and the G protein-independent signaling pathway that involves recruitment of β-arrestins [19]. May et al. have reported that PACAP induces ERK1/2 activation via two complementary mechanisms, PKC signaling and PAC1R internalization [20].

Two nonvisual β-arrestins, β-arrestin1 (arrestin2) and β-arrestin2 (arrestin3), together with two retinal isoforms, visual arrestin (arrestin1) and cone arrestin (arrestin4), belong to a superfamily of scaffolding proteins and act as multifunctional adaptor proteins that regulate GPCR signaling pathway [21–23]. β-Arrestins interact with agonist-occupied and phosphorylated GPCRs, resulting in inhibition of G-protein signaling (homologous desensitization) and further attenuate the signaling by linking receptors to clathrin-coated pits (endocytosis or sequestration). Endocytosis of GPCRs induces G-protein-independent and β-arrestin scaffold-mediated activation of several diverse signaling pathways such as mitogen-activated protein kinases (MAPKs) ERK1/2, Akt and Src [21–24].

Lyu et al. have previously identified distinct C terminus regions of PAC1R that are required for signal transduction (adenylyl cyclase and phospholipase C responses) and receptor internalization [25]. Homologous desensitization of PAC1R has been shown to be mediated by G-protein-coupled receptor kinase (GRK) 3 in human Y-79 retinoblastoma cells [26]. Merriam et al. have shown that Pitstop 2, a cell-permeable inhibitor of clathrin-mediated endocytosis, blocks PAC1R endocytosis in HEK293 cells expressing C-terminal GFP-tagged PAC1R and that PACAP/PAC1R complex endocytosis is implicated in the PACAP-induced increase of cardiac neuron excitability [27]. In pancreatic β-cell line INS-1E cells, PACAP-PAC1R-dependent signaling potentiates glucose-induced long-lasting ERK1/2 activation via a β-arrestin1-dependent pathway [28]. However, the precise roles of the two β-arrestin isoforms in PACAP-PAC1R signaling in terms of subcellular localization and subsequent prolonged ERK1/2 activation remain unclear.

In this study, we have addressed the roles of the two β-arrestin isoforms that regulates PAC1R internalization and trafficking as well as their involvement in the ERK1/2 activation.

## Materials and methods

### Drugs

PACAP (PACAP-38) was purchased from Peptide Institute (Minoh, Osaka, Japan). H89 and CMPD101 were purchased from Tocris Bioscience (Bristol, UK). D-sphingosine and Concanavarin A (ConA) were purchased from Sigma-Aldrich (St Louis, MO, USA) and Pitstop 2 (ab120687) was purchased from Abcam (Cambridge, UK).

### Vector construction

To generate the PAC1R-Halo construct, the hop1 splicing variant of human *PAC1R* cDNA (accession ID: NM_001199635) was subcloned into the pFN21A vector (HaloTag technology, Promega, Madison, WI, USA) at *Sgf*I and *Pme*I restriction sites. *Arginine vasopressin receptor* (*AVPR2*), *β-arrestin1* and *β-arrestin2* cDNAs were purchased from Flexi ORF clone book (Kazusa Genome Technologies, Kisarazu, Chiba, Japan). For construction of NanoBiT system (Promega) to detect protein-protein interaction between PAC1R and the two β-arrestin isoforms, the *PAC1R* cDNA was subcloned into the pFC36K vector to generate PAC1R-SmBiT and the *β-arrestin1* and *β-arrestin2* cDNAs were into both the pFN33K and pFC34K vectors to generate both N-terminal and C-terminal LgBiT-fused β-arrestin1 and 2 (LgBiT-β-arrestin1, LgBiT-β-arrestin2, β-arrestin1-LgBiT and β-arrestin2-LgBiT) using *Sgf*I plus *Pme*I or *EcoICR*I restriction sites. As AVPR2 is known to interact with β-arrestin2 [29], the *AVPR2* cDNA was subcloned into the pFC36K vector to be used as a positive control (AVPR2-SmBiT). The pFC36K empty vector was used for mock transfection that served as a negative control. For lentivirus vector construction, the PAC1R-SmBiT, β-arrestin1-LgBiT and LgBiT-β-arrestin2 were subcloned into the *Nhe*I- and *Not*I-digested hSyn-H2B-tdTomato-QM512B vector [30] (the QM512B vector was from the SparQ Cumate Switch system, System Biosciences, Palo Alto, CA, USA) to generate PAC1R-SmBiT-pQM512B,β-arrestin1-LgBiT-pQM512B and LgBiT-β-arrestin2-pQM512B, respectively.

### Lentivirus production

Lentiviruses were prepared as described previously [30]. Briefly, 7.8 μg of PAC1R-SmBiT-pQM512B, β-arrestin1-LgBiT-pQM512B or LgBiT-β-arrestin2-pQM512B were transfected with 23.1 μg mixed shuttle constructs to Lenti-X 293T cells (Clontech, Mountain View, CA, USA) in 15 cm dishes using polyethylenimine (Polysciences, Warrington, PA, USA). After 36 h incubation, the culture supernatant was collected. Lentiviral vector-containing media were filtered through a 0.45-μm filter and ultracentrifuged at 23,000 rpm using an SW-41 rotor (Beckman-Coulter, Brea, CA, USA) for 2 h. Lentiviral vectors were aliquoted and stored at −80°C. The titer of the lentiviral vector solution was estimated by Lenti-X qRT-PCR Titration Kit (Clontech).

### Cell culture

HEK293T cells were maintained in Dulbecco’s Modified Eagle’s Medium (DMEM) supplemented with 10% fetal bovine serum. HeLa cell line was provided by the RIKEN BRC (Tsukuba, Ibaraki, Japan), through the National Bio-Resource Project of the Ministry of Education, Culture, Sports, Science and Technology (MEXT), Japan. HeLa cells were maintained in DMEM (high glucose, GlutaMAX) supplemented with 10% fetal bovine serum (Thermo Fisher Science, Tokyo, Japan).

Primary cultures of cortical neurons were prepared as described previously [31]. Pregnant mother mice (strain, ICR) were purchased from JAPAN SLC (Shizuoka, Japan). The mice were deeply anesthetized by intraperitoneal injection of three-types mixed anesthetic agents (medetomidine hydrochloride, 0.75 mg/kg body weight; midazolam, 4 mg/kg body weight; butorphanol tartrate, 5 mg/kg body weight) and were removed fetuses (embryonic day 16). All animal care and handling procedures were performed in accordance with protocols approved by the Animal Care and Use Committee of the Graduate School of Pharmaceutical Sciences, Osaka University. Dissociated cortical cells were plated in Neurobasal medium (Invitrogen, Carlsbad, CA, USA), supplemented with B27 (Thermo Fisher Scientific) and L-glutamine (0.5 mM) at 4.0×10^5^ cells/well in 96-well dishes coated with poly-l-lysine (for NanoBiT assay), 1.2×10^6^ cells/well in 6-well dishes (for co-immunoprecipitation assay) or 2.5×10^4^ cells/well in 35 mm glass-bottom dishes (for time-lapse imaging).

All animal care and handling procedures were performed in accordance with protocols approved by the Animal Care and Use Committee of the Graduate School of Pharmaceutical Sciences, Osaka University.

### NanoBiT protein-protein interaction assay

PAC1R-SmBiT together with β-arrestin1-LgBiT or LgBiT-β-arrestin2 (each 0.5 μl per well) were used to transfect HEK293T cells in 6-well plates using Lipofectamine 2000 (Invitrogen) according to the manufacturer's protocol. After 24 h, the cells were subcultured into 96 well plates with Opti-MEM reduced Serum Medium (Life Technologies, Carlsbad, CA, USA). 30 μM G-protein coupled receptor kinase 2 and 3 (GRK2/GRK3) inhibitor CMPD101, 20 μM protein kinase A inhibitor H89 or 50 μM protein kinase C inhibitor sphingosine were pretreated for 30 min. Before PACAP treatment, Nano-Glo Live Cell Reagent (Promega) was added and baseline luminescence was measured using GloMAX discover system (Promega).

For Time-lapse imaging, PAC1R-SmBiT-pQM512B lentivirus together with β-arrestin1-LgBiT-pQM512B or LgBiT-β-arrestin2-pQM512B lentivirus were infected into HeLa cells or primary cultured cortical neurons of 3 days in vitro (DIV 3). After Nano-Glo Live Cell Reagent (Promega) was added, the luminescence was measured using Olympus LV200 bioluminescence imager (Olympus, Tokyo, Japan) and the time-lapse images were processed either using Metamorph software (Molecular Devices Japan, Tokyo, Japan) or the ImageJ software (https://imagej.net/). To assess the luminescence intensity at the plasma membrane and the cytoplasm, we defined the shape of the whole-cell (region of interest (ROI) A) and the cytoplasm region (ROI B) by reducing the size by 7 pixels and determined the luminescence in the both ROIs. The amount of luminescence at the vicinity of the plasma membrane was defined by subtracting the amount of luminescence in ROI A by that in ROI B. All analyses were performed in a blind manner.

### β-arrestin silencing

For siRNA-induced silencing of β-arrestins, β-arrestin1 (#6218S; Cell Signaling Technology, Danvers, MA, USA), β-arrestin2 (#sc-29743; Santa Cruz Biotechnology, Dallas, TX) or control siRNA (#6568S; Cell Signaling Technology) each at 25 mM were transfected using Lipofectamine RNAiMAX (Invitrogen) according to the manufacturer's protocol.

### PAC1R internalization

Internalization of PAC1R was quantitatively assessed using HaloTag technology according to the manufacturer's protocol (Promega). HEK293T cells were transfected with PAC1R-Halo-expressing vector together with β-arrestin1 siRNA, β-arrestin2 siRNA or control siRNA; the cells were then labeled with the cell-impermeable Alexa Fluor 488 ligand (Promega) in Opti-MEM for 15 min at 37°C. Clathrin-mediated endocytosis inhibitor, 250 μg/ml ConA or 15 μM Pitstop2, were pretreated for 30 min. After 30 min, the cells were stimulated with 1 nM PACAP or saline, and were then washed with phosphate-buffered saline and fixed in 4% paraformaldehyde. The cell images were obtained using FV1000D confocal microscope (Olympus) in a sequential mode and membrane protein internalization was quantified using the ImageJ software. To assess the internalization ratio of PAC1R, we defined the shape of the whole-cell (ROI A) and the cytoplasm region (ROI B) by reducing the size by 5–10 pixels and determined the fluorescence in the both ROIs. The internalization ratio (%) was defined by dividing the amount of luminescence in ROI B by that in ROI A. All analyses were performed in a blind manner.

### Antibody

Commercially available antibodies used were as follows: anti-ERK1/2 antibody (Cell Signaling Technology), anti-phospho-ERK1/2 antibody (Cell Signaling Technology), anti-β-arrestin1/2 antibody (Cell Signaling Technology), anti-PAC1R antibody (Abcam), normal mouse IgG (Millipore, Darmstadt, Germany) and normal rabbit IgG (Millipore). Horseradish peroxidase-conjugated anti-rabbit IgG and anti-mouse IgG were purchased from Cappel (Cochranville, PA, USA).

### Chemical crosslinking and co-immunoprecipitation

For the detection of PACAP-induced association of PAC1 and β-arrestin1 or β-arrestin2, covalent protein cross-linking with a chemical crosslinker, Dithiobis succinimidylpropionate (DPS; Thermo Fisher Scientific) was used as described previously [32, 33]. HEK293T cells were washed with PBS containing 10 mM Hepes (pH 7.4) and were incubated with 2.5 mM DSP for 30 min at room temperature. The reactions were quenched by the addition of 0.1 ml of 1 M Tris (pH 7.5). For prevention of co-elution of IgG fragments, the immobilized antibodies were covalently cross-linked to protein G-Sepharose beads. Antibody-coupled protein G-Sepharose beads were resuspended in 5 mM BS3 (Thermo Fisher Scientific) and incubated for 30 min at room temperature. Reactions were quenched by the addition of 0.1 ml of 1 M Tris (pH 7.5). Cells were lysed in RIPA buffer and the resultant lysates were incubated with the indicated antibody-coupled protein G-Sepharose beads for 2 h at 4°C. The beads were then washed three times with RIPA buffer and suspended in SDS sample buffer.

### Western blotting

HEK293T cells were lysed in RIPA buffer (50 mM Tris-HCl, pH 7.4, 150 mM NaCl, 1 mM EDTA, 0.1% NP-40, 0.5% DOC, 0.1% SDS) and the resultant lysates were separated by SDS-PAGE with 10% polyacrylamide gels and transferred electrophoretically onto polyvinylidene fluoride membranes (Millipore). After blocking with 2% BSA in TNE buffer, the membranes were incubated with an anti-ERK1/2 antibody (1:1,000 dilution), anti-phospho-ERK1/2 antibody (1:1,000 dilution), PAC1R antibody (1:1,000 dilution) or anti-β-arrestin1/2 antibody (1:1,000 dilution) overnight at 4°C. After incubation with a horseradish peroxidase-conjugated anti-rabbit IgG or anti-mouse IgG (1:2,000 dilution) secondary antibody for 1 h at room temperature, proteins were detected by chemiluminescence and visualized with an ImageQuant LAS 4000 system (GE Healthcare, Little Chalfont, UK). For quantification, the bands for specific immune-complexes were analyzed using the ImageJ software.

### Statistical analysis

Experimental data were analyzed using one-way or two-way analysis of variance (ANOVA). Fisher-PLSD *post hoc* tests were also performed after significant main effects for drug, time or luminescence intensity were observed. The criterion for statistical significance was p < 0.05. Statistical analyses were performed using Stat View software (version 5.0; SAS Institute, Cary, NC, USA).

## Results

### PAC1R interacts with β-arrestin1 and β-arrestin2

To examine whether PAC1R directly couples with β-arrestin1 and β-arrestin2, we used NanoBiT (NanoLuc Binary Technology; Promega), which can assess intracellular proteinprotein interactions by structural complementation of luciferase. HEK293T cells were transiently transfected with various combinations of plasmid vectors that express SmBiT (the positive control AVPR2-SmBiT, the negative control pFC36K or PAC1R-SmBiT) and LgBiT (N- or C-terminal LgBiT-fused β-arrestin1 and 2).

**Fig 1:**
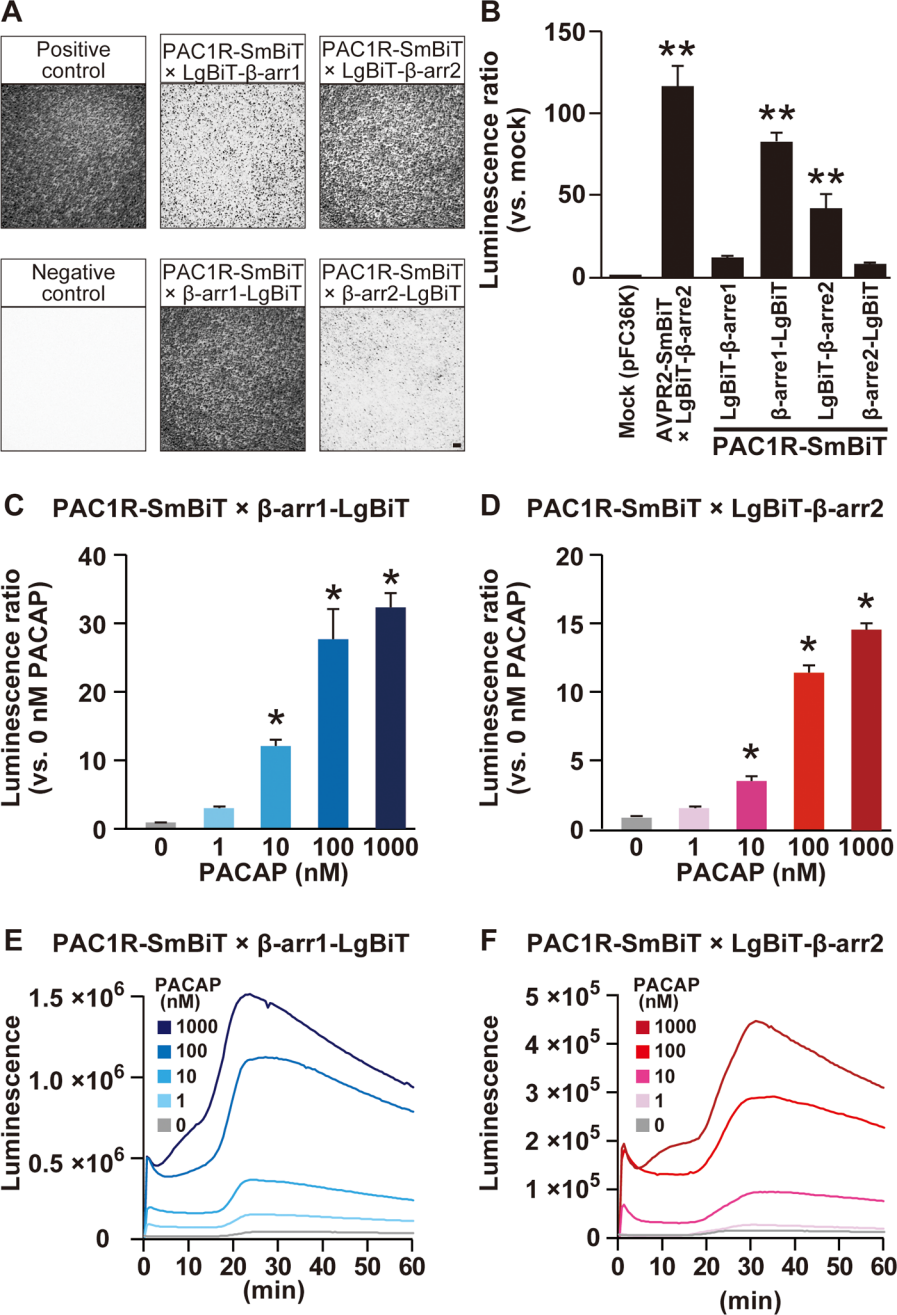
PAC1R interacts with and β-arrestin2 with similar PACAP-dose and time dependency. HEK293T cells were transfected with the indicated combinations of plasmid vectors. **(A)** Representative images of NanoBiT luminescence 15 min after addition of 100 μM PACAP. Scale bar, 1 mm. **(B)** The ratios of luminescence intensity compared with cells mock transfected with pFC36K empty vector. AVPR2-SmBiT serves as positive control. **p < 0.01 vs. mock, one-way ANOVA followed by Fisher-PLSD test. **(C and D)** PACAP-dose dependent increase in luminescence accumulation intensity which was determined 60 min after PACAP addition. *p < 0.05 vs. 0 nM PACAP, one-way ANOVA followed by Fisher-PLSD test. **(E and F)** Time course of changes in luminescence intensity for 60 min after addition of the indicated concentrations of PACAP. β-arr1, β-arrestin1; β-arr2, β-arrestin2. Values are mean ± SEM **(B–D)** or means **(E and F)** of three independent experiments each conducted in duplicate.

The positive control AVPR2-SmBiT produced luciferase luminescence more than 100-fold stronger than the negative control mock transfection (Fig 1A and 1B). C-terminal LgBiT-fused β-arrestin1 (β-arrestin1-LgBiT) gave stronger luminescence than N-terminal LgBiT-fused β-arrestin1 (LgBiT-β-arrestin1), while N-terminal LgBiT-fused β-arrestin2 (LgBiT-β-arrestin2) gave stronger luminescence than C-terminal LgBiT-fused β-arrestin1 (β-arrestin2-LgBiT) in HEK293T cells co-expressed with PAC1R-SmBiT and stimulated with 100 nM PACAP (Fig 1A and 1B). We therefore used C-terminal fused β-arrestin1-LgBiT and N-terminal fused LgBiT-β-arrestin2 to examine PAC1R and β-arrestin coupling and translocation. PACAP increased luminescence with very similar dose-dependency (Fig 1C and 1D) and time-course (Fig 1E and 1F) in β-arrestin1-LgBiT and LgBiT-β-arrestin2 in HEK293T cells co-expressed with PAC1R-SmBiT. The luminescence increased within 2 min and lasted at least 60 min for either β-arrestin1 and β-arrestin2. To further investigate whether β-arrestin1 and β-arrestin2 interact with PAC1R, we also performed co-immunoprecipitation experiments in HEK293T cells. We found that PAC1R interacted with β-arrestin1 and β-arrestin2 in PACAP-treated HEK293T cells (S1 Fig). The GRK2/GRK3 inhibitor CMPD101 similarly inhibited the PACAP-induced PAC1R interaction with β-arrestin1-LgBiT and LgBiT-β-arrestin2 (Panels A and B in S2 Fig). These two interactions were not affected by the protein kinase A inhibitor H89 or the protein kinase C inhibitor D-sphingosine (Panels C–F in S2 Fig).

### Time-lapse cell imaging of PAC1R and β-arrestin coupling and translocation in HeLa cells and primary cultured cortical neurons

**Fig 2:**
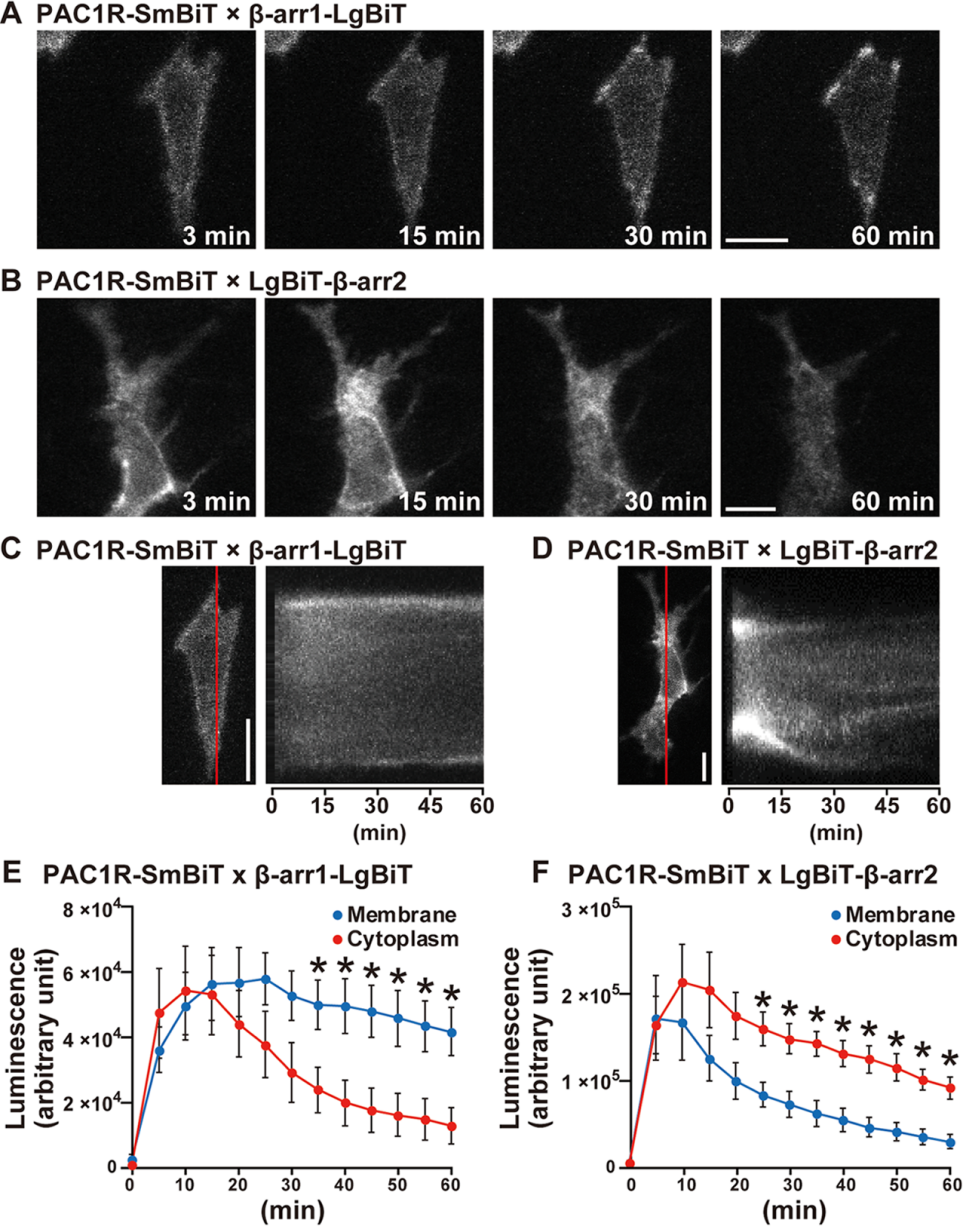
Time-lapse cell imaging showing PAC1R and β-arrestin coupling and translocation in HeLa cells. HeLa cells were transfected with the indicated combinations of plasmid vectors. **(A and B)** Representative images of NanoBiT luminescence at 3, 15, 30 and 60 min after stimulation with 1 μM PACAP. **(C and D)** Representative time-dependent changes of line-scan images for 60 min after stimulation with 1 μM PACAP. **(E and F)** Time course of changes in luminescence intensity at the vicinity of the plasma membrane (membrane) and the cytoplasm for 60 min after stimulation with 1 μM PACAP in HeLa cells. Scale bars, 10 μm. β-arr1, β-arrestin1; β-arr2, β-arrestin2. Values are mean ± SEM (n = 3–5). *p < 0.05 vs. cytoplasm, repeated measure two-way ANOVA followed by Fisher-PLSD test. See also S1 and S2 Movies.

We examined whether β-arrestin1 and β-arrestin2 are implicated in the PAC1R internalization by time-lapse cell imaging to visualize PAC1R and the two β-arrestin isoforms coupling and translocation in HeLa cells. At as early as 3 min after 1 μM PACAP stimulation, the NanoBiT signals from β-arrestin1 and β-arrestin2 (β-arrestin1-LgBiT and LgBiT-β-arrestin2) co-expressed with PAC1R-SmBiT were clearly detectable and localized around the plasma membrane and cytoplasm. At 15 min or later, however, the signal from PAC1R-β-arrestin1 complex remained localized around the plasma membrane, whereas that from PAC1R-β-arrestin2 complex localized predominantly at the cytoplasm (Fig 2; S1 and S2 Movies). Time-dependent changes of line-scan images and quantitative analysis of time course changes in luminescence at the vicinity of the plasma membrane and the cytoplasm showed that PAC1R-β-arrestin2 complex was translocated from the plasma membrane to the cytoplasm and unevenly clustered, while PAC1R-β-arrestin1 complex remained around the plasma membrane (Fig 2C–2F) [β-arrestin1, repeated measures two-way ANOVA, time effect, *F*_(1, 96)_ = 22.0, *p* < 0.0001; time × luminescence intensity effect, *F*_(1, 96)_ = 6.20, *p* < 0.0001; post hoc, p < 0.05; β-arrestin2, repeated measures two-way ANOVA, time effect, *F*_(1, 96)_ = 18.2, *p* < 0.0001; time × luminescence intensity effect, *F*_(1, 96)_ = 1.62, *p* = 0.099; post hoc, p < 0.05].

**Fig 3:**
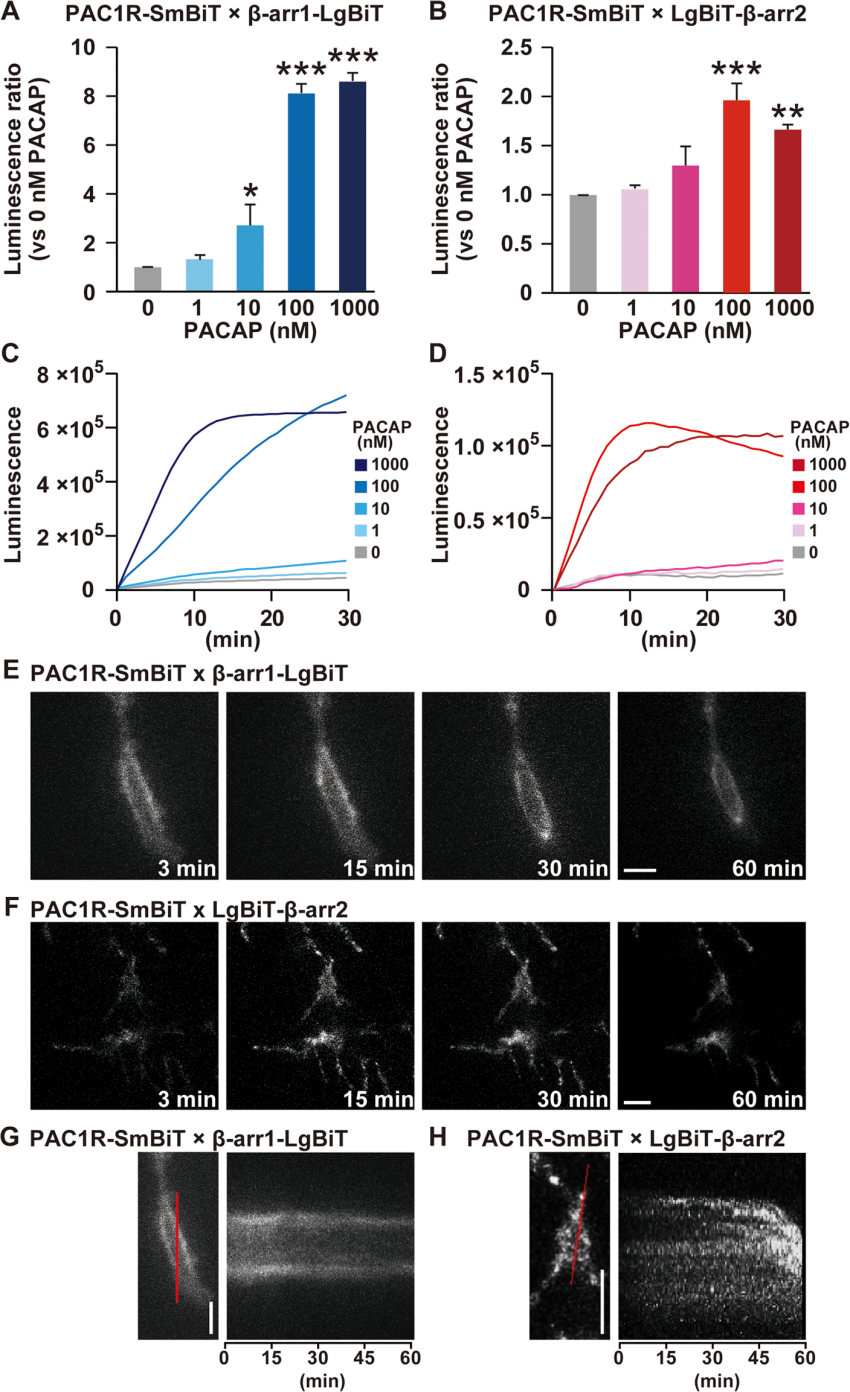
PAC1R and β-arrestin coupling and translocation in primary cultured cortical neurons. Primary cultured cortical neurons were infected with the indicated combinations of lentiviruses. **(A and B)** PACAP-dose dependent increase in NanoBiT luminescence accumulation intensity which was determined 60 min after PACAP addition. **(C and D)** Time course of changes in luminescence intensity for 60 min after addition of the indicated concentrations of PACAP. **(E and F)** Representative images of NanoBiT luminescence at 3, 15, 30 and 60 min after stimulation with 1 μM PACAP. **(G and H)** Representative time-dependent changes of line-scan images for 60 min after stimulation with 1 μM PACAP. Scale bars, 10 μm. β-arr1, β-arrestin1; β-arr2, β-arrestin2. Values are mean ± SEM **(A and B)** or mean **(C and D)** of three independent experiments each conducted in duplicate. *P < 0.05, **p < 0.001 and ***p < 0.001 vs. 0 nM PACAP, one-way ANOVA followed by Fisher-PLSD test. See also S3 and S4 Movies.

To confirm the PAC1R and β-arrestin coupling in more biologically relevant cells, we also used mouse primary cultured cortical neurons. PACAP increased the luciferase luminescence with similar dose-dependency and time-course in cortical neurons infected with PAC1R-SmBiT-pQM512B lentivirus in combination with either β-arrestin1-LgBiT-pQM512B or LgBiT-β-arrestin2-pQM512B lentiviruses (Fig 3A–3D). These dose- and time-dependent changes were virtually similar as those observed in HEK293T cells (Fig 1C–1F). In addition, time-lapse cell imaging and time-dependent changes of line-scan images of these cortical neurons were very similar to those observed in HEK293T cells (Fig 3E–3H; S3 and S4 Movies). These results suggest that PAC1R is internalized via distinct pathways in complex with β-arrestin1 and β-arrestin2.

### Silencing of β-arrestin2, but not β-arrestin1, inhibits PAC1R internalization

**Fig 4:**
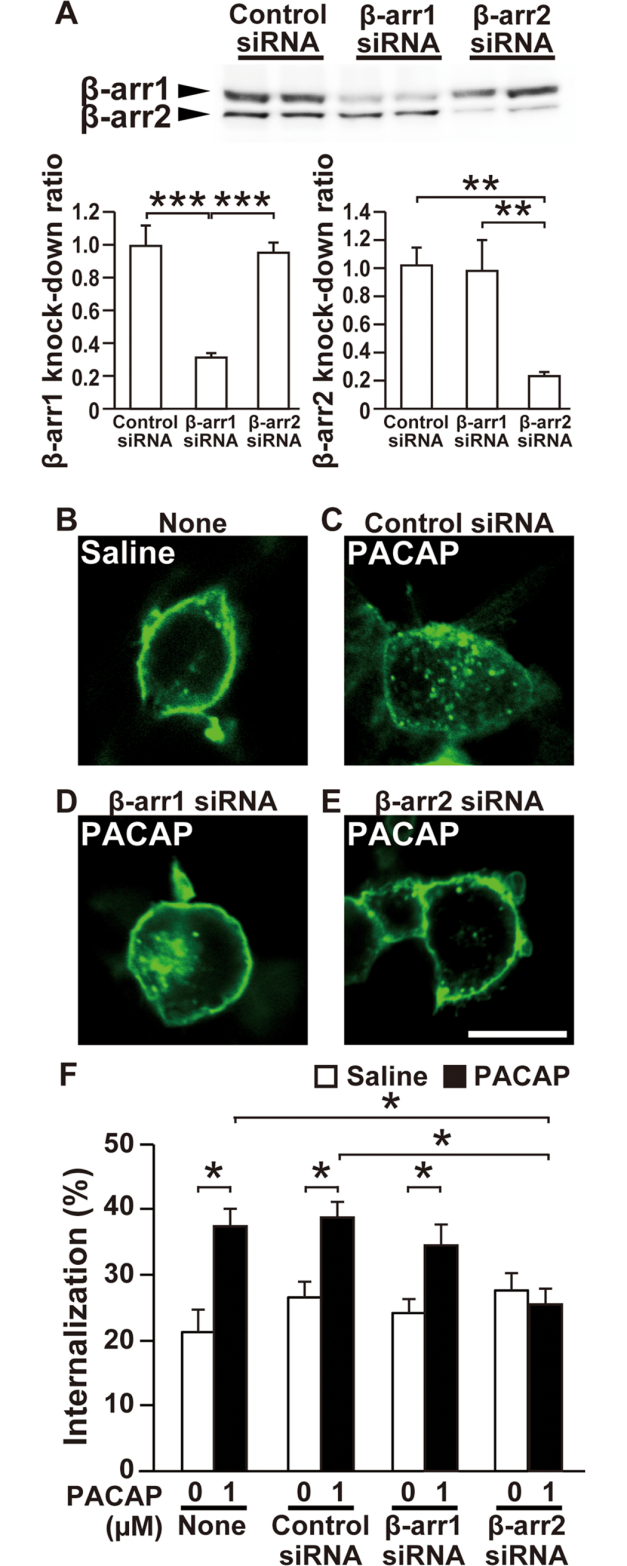
Differential effects of β-arrestin1 and β-arrestin2 siRNA on PAC1R internalization. **(A)** Knockdown of endogenous β-arrestin1 and β-arrestin2 in HEK293T cells. The upper images, representative images of western blots. Values are mean ± SEM of three independent experiments. **p < 0.01 and ***p < 0.001, two-way ANOVA followed by Fisher-PLSD test. **(B–E)** Representative images of HEK293T cells that were transfected with PAC1R-Halo and the indicated siRNA, labeled with Alexa Fluor 488 HaloTag ligand and treated with 1 μM PACAP or saline for 30 min. **(F)** Quantification of PAC1R-Halo internalization. Scale bar, 10 μm. β-arr1, β-arrestin1; β-arr2, β-arrestin2. Values are mean ± SEM of 60 cells obtained from three independent experiments. *p < 0.05 and **p < 0.01, two-way ANOVA followed by Fisher-PLSD test.

We examined whether β-arrestin1 and β-arrestin2 are implicated in the PAC1R internalization. The β-arrestin1 and β-arrestin2 siRNAs effectively decreased the respective β-arrestin levels to less than 35% in HEK293T cells (Fig 4A). The internalization of PAC1R was assessed in HEK293T cells using PAC1R-Halo overexpression. We labeled only cellular surface PAC1R-Halo proteins with the cell- impermeable Alexa Fluor 488 HaloTag ligand and measured the signal ratio of internalized PAC1R vs. total PAC1R in each single cell after PACAP treatment. In the absence of PACAP, PAC1R-Halo was located mainly on the plasma membrane and the internalized PAC1R-Halo was approximately 20% (Fig 4B and 4F). After stimulation with 1 μM PACAP for 30 min, punctate Halo-PAC1R signals increased in the cytoplasm and the internalized PAC1R-Halo amounted to 37% (Fig 4F). The silencing of β-arrestin2 blocked PACAP-induced increase in PAC1R-Halo internalization, whereas the silencing of β-arrestin1 as well as control siRNA showed no significant effect on PAC1R-Halo internalization (Fig 4C–4F). Moreover, an inhibitor of clathrin-mediated endocytosis, ConA significantly inhibited the PACAP-induced PAC1R-Halo internalization in HEK293T cells (Panels A–E in S3 Fig). Likewise, other inhibitor of clathrin-mediated endocytosis, Pitstop2 almost completely inhibited the PACAP-induced PAC1R-Halo internalization (Panels A–E in S3 Fig).

### Silencing of β-arrestin2, but not β-arrestin1, inhibits prolonged ERK1/2 activation

**Fig 5:**
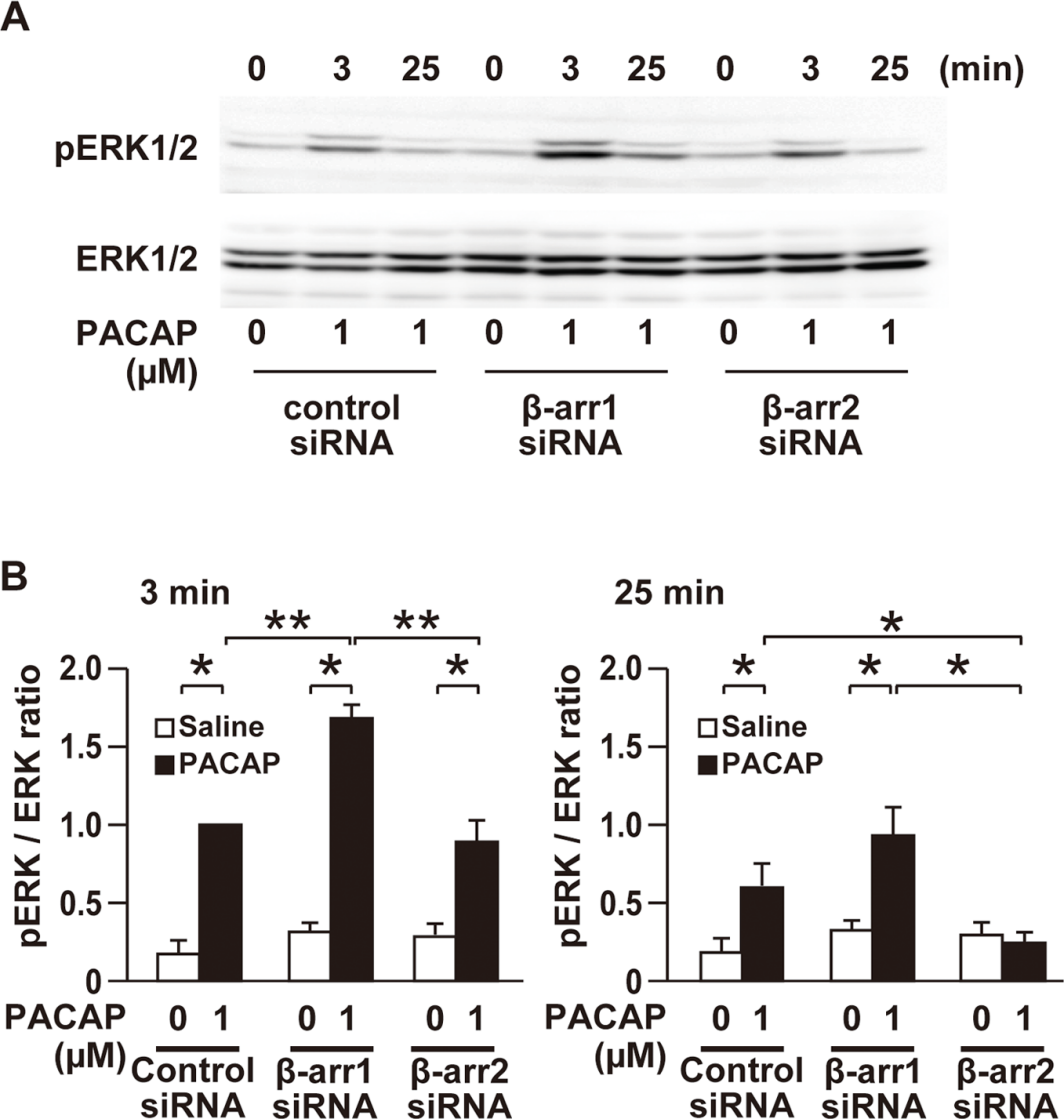
Differential effects of silencing of β-arrestin1 and β-arrestin2 on PACAP-induced ERK1/2 activation. **(A)** Representative images of western blots for total and phosphorylated ERK1/2 in HEK293T cells transfected with the indicated siRNA and treated with 1 μM PACAP or saline for 3 or 25 min. **(B)** Quantification of ERK1/2 activation by normalizing phosphorylated ERK1/2 to total ERK1/2 levels analyzed by western blotting. β-arr1, β-arrestin1; β-arr2, β-arrestin2. Values are mean ± SEM of three independent experiments. *p < 0.05 and **p < 0.01, two-way ANOVA followed by Fisher-PLSD test.

We addressed whether β-arrestin2 is also implicated in the PACAP-induced ERK1/2 activation in HEK293T cells (Fig 5). In the absence of PACAP, phosphorylated ERK1/2 levels were not significantly changed by the silencing of β-arrestin1 or β-arrestin2. PACAP (1 μM)-increased ERK1/2 phosphorylation at an early time point (3 min) was not changed by the β-arrestin2 silencing but rather increased by the β-arrestin1 silencing compared with the control siRNA. At a later time point (25 min), phosphorylated ERK1/2 levels were decreased by the β-arrestin2 silencing while they were increased by the β-arrestin1 silencing compared with the control siRNA. To examine whether PACAP-induced ERK1/2 phosphorylation is required for the PAC1R internalization, the cells were pretreated with 250 μg/ml ConA or 15 μM Pitstop2 before treatment with 1 μM PACAP. Pretreatment with ConA significantly decreased the PACAP-increased ERK1/2 phosphorylation (Panels F and G in S3 Fig). Likewise, Pitstop2 almost completely inhibited the PACAP-increased ERK1/2 phosphorylation (Panels F and G in S3 Fig).

## Discussion

In the present study, we have investigated PAC1R internalization that depends on β-arrestin1 and β-arrestin2 using the NanoBiT system, HaloTag technology and siRNA-mediated silencing of endogenous β-arrestins in HEK293T cells, HeLa cells and primary cultured cortical neurons. For this purpose, we used time-lapse luminescence microscopy and processed the images to detect intracellular translocation. In addition, we examined the roles of β-arrestin1 and β-arrestin2 involved in the PACAP-stimulated PAC1R-mediated prolonged ERK1/2 activation relevant to the PAC1R internalization.

Here we showed that PACAP-stimulated PAC1R interacted with β-arrestin1 and β-arrestin2 with similar PACAP dose- and time-dependency and that subcellular distribution of the PAC1R and β-arrestin2 complex changed time-dependently, whereas the complex with β-arrestin1 seemed to remain at the cell surface regions. We also showed that silencing of β-arrestin2 significantly reduced PACAP-induced PAC1R internalization and prolonged ERK1/2 activation, but silencing of β-arrestin1 did not affect PAC1R internalization and rather increased ERK1/2 phosphorylation. Moreover, we found evidence suggesting that PACAP-induced ERK1/2 phosphorylation was dependent on PAC1R internalization using inhibitors of clathrin-mediated endocytosis, ConA and Pitstop2. Taken together, these results suggest that β-arrestin2 but not β-arrestin1 is critically involved in the PAC1R endocytosis and subsequent activation of the ERK1/2 signalosome. These findings contribute to the mechanistic understanding of previous studies showing that PAC1R endocytosis is implicated in the PACAP-increased ERK1/2 activation [20, 27]. However, the residual β-arrestin1 after β-arrestin1 knockdown may be enough for the PAC1R endocytosis and subsequent ERK1/2 activation. Further studies with genome editing technology are needed for elucidating the precise functional diversity of β-arrestin1 and β-arrestin2 in the PACAP signaling.

In recent studies, accumulating evidence suggests that β-arrestin1 and β-arrestin2 have distinct roles in various GPCR signaling events [34, 36–39]. In cardiomyocytes, β-arrestin1 desensitizes β1AR cAMP-dependent pro-contractile signaling and β-arrestin2 interacts with SERCA2a and affects cardiac SERCA2a SUMOylation and activity in a β1AR-dependent manner [36]. In melanocytes, although both β-arrestin1 and β-arrestin 2 interact with the melanocortin1 receptor (MC1R), β-arrestin2, but not β-arrestin1, can specifically induce ERK1/2 activation and MC1R internalization [37]. In the present study, we found that PAC1R interacted with β-arrestin1 and β-arrestin2 in PACAP-treated HEK293T cells by co-immunoprecipitation experiments. Our results using NanoBiT protein-protein interaction assay suggested that β-arrestin1 interacted with the PAC1R via its N-terminus, whereas β-arrestin2 interacted with the PAC1R via its C-terminus. The precise mechanism for the differential interaction of PAC1R and β-arrestins is currently unknown. Although β-arrestin1 and β-arrestin2 have structural similarity (more than 70% identity), there was a considerable structural diversity in the C-terminus [34, 35]. Furthermore, recent studies showed that β-arrestin1 and β-arrestin2 have distinct roles in various GPCR signaling events [34, 36–39]. These structural and functional diversities might account for the differential interaction of PAC1R and β-arrestins.

In the current study, we observed that silencing of β-arrestin2 inhibits prolonged ERK1/2 activation (25 min after PACAP stimulation), whereas, in contrast, β-arrestin1 siRNA increases ERK1/2 activation (3 min after PACAP stimulation) in HEK293T cells. The same reciprocal activity of the two β-arrestin isoforms on ERK1/2 activation has been demonstrated for the angiotensin II type 1A receptor-mediated activation of ERK1/2, which is increased under β-arrestin1 silencing but is eliminated under β-arrestin2 silencing in HEK-293 cells [40]. In contrast to our current results, Gupte et al. have demonstrated that PACAP-induced ERK1/2 activation mediated by PAC1R (the hop1 and hop2 splicing variants) is abolished by β-arrestin1 silencing, but not by β-arrestin2 silencing, in HEK293T cells [41]. The reason for the opposing results in the β-arrestin isoform-dependent regulation of ERK1/2 activation is currently unknown. The discrepancies might be explained by the differential experimental conditions; Gupte et al. used the overexpressed PAC1R and β-arrestins, while we use endogenous PAC1R and β-arrestins in this study. The residual β-arrestin after β-arrestin knockdown should also be taken into account. May et al. demonstrated that PACAP-stimulated ERK phosphorylation is diminished by Pitstop 2 and dynasore, inhibitors of clathrin-mediated endocytosis [20]. They have also demonstrated that PACAP-stimulated protein kinase C signaling contributes to ERK phosphorylation [20]. In the present study we observed that a protein kinase C inhibitor, D-sphingosine, shows no effect on the interaction of PAC1R with either β-arrestin1 or β-arrestin2 (Panels C and D in S2 Fig). These results are consistent with those reported by May et al. [20] and suggest that protein kinase C may be required for PACAP-stimulated ERK1/2 phosphorylation downstream of PAC1R internalization. Moreover, we found that treatment with inhibitors of clathrin-mediated endocytosis, ConA and Pitstop2, reduces PACAP-stimulated ERK1/2 activation. These results suggested that PACAP-induced ERK1/2 phosphorylation was dependent on PAC1R internalization. We also observed that β-arrestin2 plays a role in PAC1R internalization using β-arrestin2 siRNA. These results indicate that PAC1R-activation generally leads to β-arrestin-scaffolding of the ERK1/2 cascade and enhances ERK1/2 activity, while the two β-arrestin isoforms involved in this pathway depend on tissues and physiological conditions. The present results suggest that β-arrestin1 and β-arrestin2 might be involved in fine and precise tuning of the PAC1R signaling pathways.

The finding of the β-arrestin-mediated signaling implicated signaling-biased ligands that favor G-protein signaling over arrestin recruitment or vice versa, which proposes therapeutic potential [21, 24, 42–44]. In the case of antipsychotic drugs, G protein- and β-arrestin2-biased functional selectivity at dopamine D_2_ receptor is expected to have the potential to correct both positive and cognitive symptoms of schizophrenia [45]. Because PACAP and VIP signals might be related to psychiatric and neurological disorders, the present observations as well as the assay systems generated are expected to contribute to therapeutic drug development.

## Supporting information

S1 Fig The interactions between PAC1R and β-arrestin1/2 in PACAP-treated HEK293T cells were detected by co-immunoprecipitation experiments. HEK293T cells were stimulated with 1 μM PACAP for 15 min. **(A)** The immunoprecipitates with antibodies indicated were subjected to immunoblotting with anti-PAC1R and anti-β-arrestin1/2 antibodies.
(TIF)

S2 Fig PACAP-induced interaction between PAC1R and β-arrestin1/2 is inhibited by CMPD101. HEK293T cells were transfected with the indicated combinations of plasmid vectors. The G-protein coupled receptor kinase 2 and 3 (GRK2/GRK3) inhibitor CMPD101 at 30 μM **(A, B)**, the protein kinase A inhibitor H89 at 20 μM **(C, D)** or the protein kinase C inhibitor sphingosine at 50 μM **(E, F)** were pretreated for 30 min and PACAP at the indicated concentrations for 60 min before the measurement of luminescence. β-arr1, β-arrestin1; β-arr2, β-arrestin2. Values are mean ± SEM of three independent experiments. *p < 0.05, two-way ANOVA followed by Fisher-PLSD test.
(TIF)

S3 Fig PACAP-induced PAC1 internalization and ERK1/2 activation are inhibited by ConA and Pitstop2. **(A-D)** Representative images of HEK293T cells transfected with PAC1R-Halo. Cells were treated with clathrin-mediated endocytosis inhibitors, ConA (250 μg/ml) **(C)** or Pitstop2 (15 μM) **(D)** were pretreated for 30 min, labeled with Alexa Fluor 488 HaloTag ligand and treated with 1 μM PACAP or saline for 30 min. Scale bar, 10 μm. **(E)** Quantification of PAC1R-Halo internalization. Scale bar, 10 μm. Values are mean ± SEM of 60 cells obtained from three independent experiments. *p < 0.05, two-way ANOVA followed by Fisher-PLSD test. **(F)** Representative images of western blots for total and phosphorylated ERK1/2 in HEK293T cells treated with the endocytosis inhibitors and 1 μM PACAP or saline for 25 min. **(G)** Quantification of phosphorylated ERK1/2 activation by normalizing phosphorylated ERK1/2 levels to total ERK1/2 levels analyzed by western blotting. Values are mean ± SEM of three independent experiments. *p < 0.05, two-way ANOVA followed by Fisher-PLSD test.
(TIF)

S1 Movie Time-lapse cell imaging of PAC1R and β-arrestin1 interaction in HeLa cells. (MP4)

S2 Movie Time-lapse cell imaging of PAC1R and β-arrestin2 interaction in HeLa cells. (MP4)

S3 Movie Time-lapse cell imaging of PAC1R and β-arrestin1 interaction in the primary cultured cortical neurons. (MP4)

S4 Movie Time-lapse cell imaging of PAC1R and β-arrestin2 interaction in the primary cultured cortical neurons. (MP4)
