# Supplementary figures and images for: β-Arrestin1 and 2 differentially regulate PACAP-induced PAC1 receptor signaling and trafficking
Source: PLoS One. 2018 May 7;13(5):e0196946. doi: 10.1371/journal.pone.0196946 (PMC5937772; doi:10.1371/journal.pone.0196946)

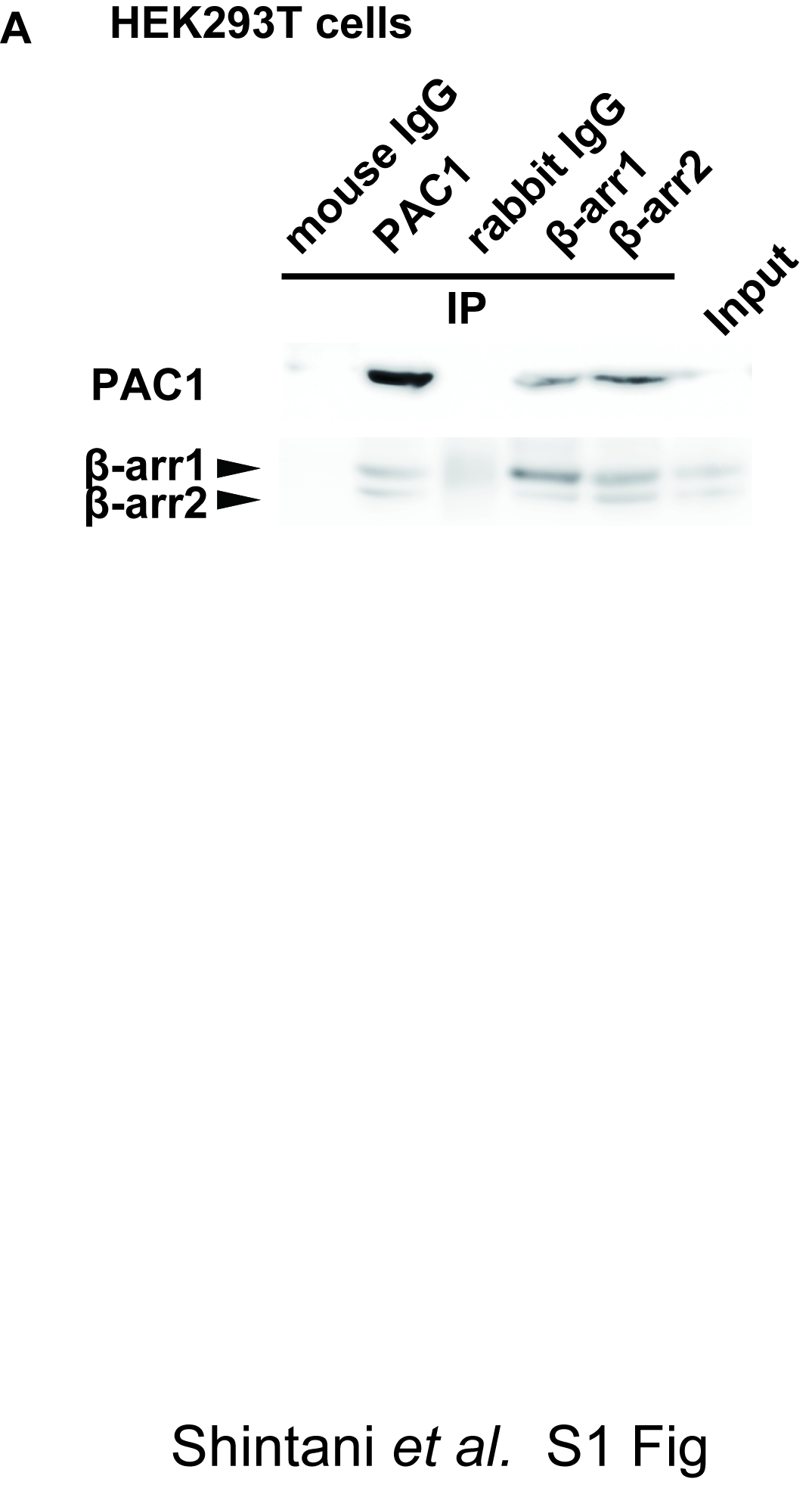

Supplement: S1 Fig — HEK293T cells were stimulated with 1 μM PACAP for 15 min. (A) The immunoprecipitates with antibodies indicated were subjected to immunoblotting with anti-PAC1R and anti-β-arrestin1/2 antibodies. (TIF) [file pone.0196946.s001.tif]

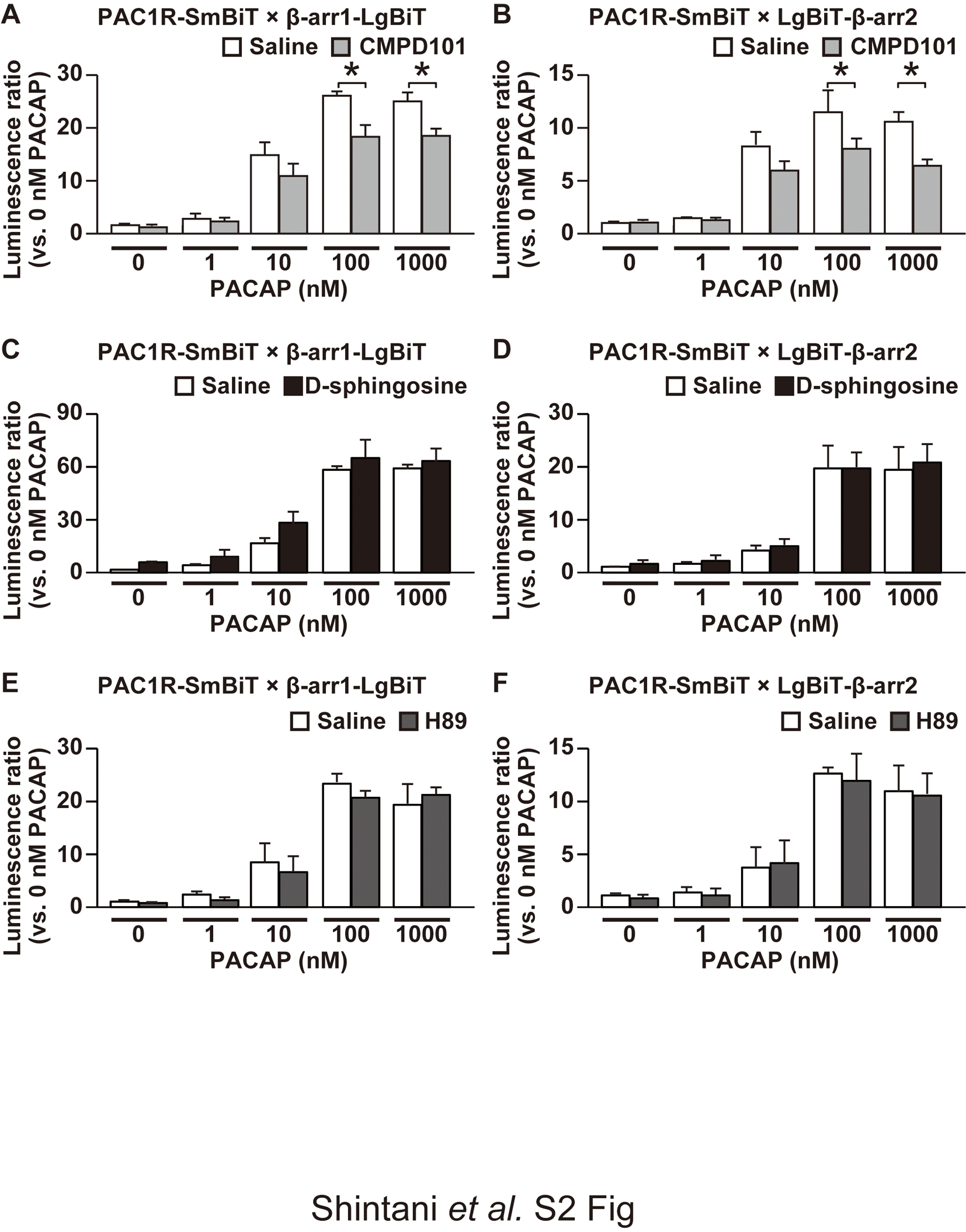

Supplement: S2 Fig — HEK293T cells were transfected with the indicated combinations of plasmid vectors. The G-protein coupled receptor kinase 2 and 3 (GRK2/GRK3) inhibitor CMPD101 at 30 μM (A, B), the protein kinase A inhibitor H89 at 20 μM (C, D) or the protein kinase C inhibitor sphingosine at 50 μM (E, F) were pretreated for 30 min and PACAP at the indicated concentrations for 60 min before the measurement of luminescence. β-arr1, β-arrestin1; β-arr2, β-arrestin2. Values are mean ± SEM of three independent experiments. *p < 0.05, two-way ANOVA followed by Fisher-PLSD test. (TIF) [file pone.0196946.s002.tif]

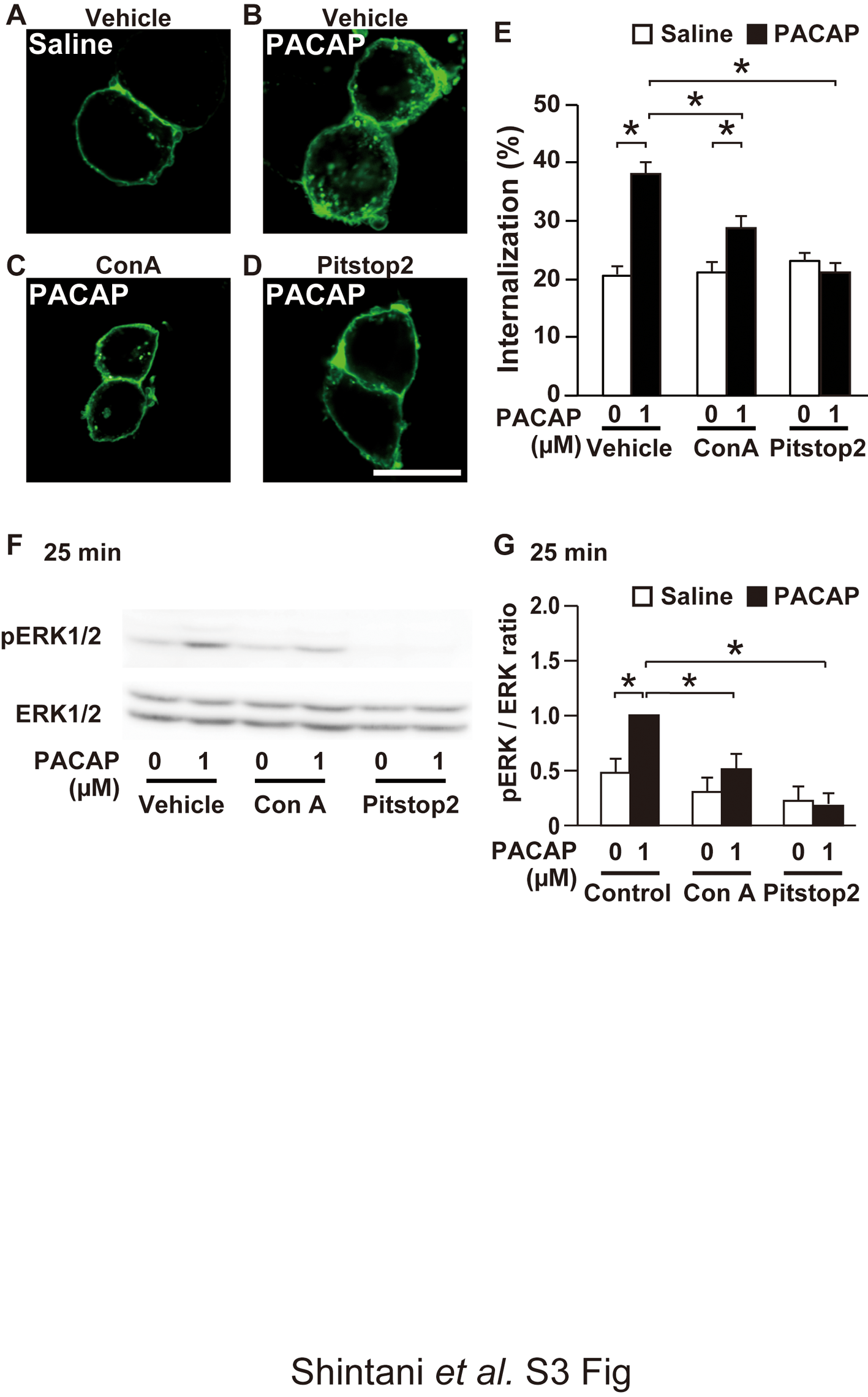

Supplement: S3 Fig — (A-D) Representative images of HEK293T cells transfected with PAC1R-Halo. Cells were treated with clathrin-mediated endocytosis inhibitors, ConA (250 μg/ml) (C) or Pitstop2 (15 μM) (D) were pretreated for 30 min, labeled with Alexa Fluor 488 HaloTag ligand and treated with 1 μM PACAP or saline for 30 min. Scale bar, 10 μm. (E) Quantification of PAC1R-Halo internalization. Scale bar, 10 μm. Values are mean ± SEM of 60 cells obtained from three independent experiments. *p < 0.05, two-way ANOVA followed by Fisher-PLSD test. (F) Representative images of western blots for total and phosphorylated ERK1/2 in HEK293T cells treated with the endocytosis inhibitors and 1 μM PACAP or saline for 25 min. (G) Quantification of phosphorylated ERK1/2 activation by normalizing phosphorylated ERK1/2 levels to total ERK1/2 levels analyzed by western blotting. Values are mean ± SEM of three independent experiments. *p < 0.05, two-way ANOVA followed by Fisher-PLSD test. (TIF) [file pone.0196946.s003.tif]
